# Impact of limb length discrepancy on functional outcome in total knee arthroplasty patients: a prospective cohort study

**DOI:** 10.1186/s42836-022-00123-w

**Published:** 2022-06-02

**Authors:** Siddharth Satyakam Pradhan, Sujit Kumar Tripathy, Mantu Jain, Hrudeswar Behera, Sandeep Velagada, Anand Srinivasan

**Affiliations:** Dept. of Orthopedics, All India Institute of Medical Sciences, Bhubaneswar-751019, India

## Abstract

**Background:**

Limb length alteration following total knee arthroplasty (TKA) has been under-reported. Few studies have shown a significant association between limb length discrepancy (LLD) and poor functional outcome. This prospective study evaluated the impact of radiographic and perceived LLD on functional outcome in TKA. The variables affecting LLD were also evaluated.

**Methods:**

The preoperative and postoperative limb lengths of TKA patients (112 knees, 81 patients, KL grade ≥ 3) were measured in full-length digital radiographs. The Hip-Knee-Ankle (HKA) angles were also measured. The functional outcome (Western Ontario and McMaster Universities Arthritis Index) and perception about LLD were evaluated after six months.

**Results:**

The mean preoperative radiographic LLD in the unilateral and bilateral TKA groups was 0.75 cm ± 0.60 cm and 0.58 cm ± 0.52 cm (*P* = 0.197), respectively. Similarly, postoperative LLD was 0.76 cm ± 0.85 cm in the unilateral group and was 0.59 cm ± 0.92 cm (*P* = 0.402) in the bilateral group. Only 19.7% of patients had postoperative radiographic LLD of ≥ 10 mm, and 80.2% of patients had LLD of < 10 mm. The functional outcome was significantly affected when LLD exceeded 10 mm (correlation coefficient 0.54, *P* < 0.001). Linear regression analysis revealed no significant effects of age, sex, height, weight, BMI, preoperative LLD and difference in deformity between the limbs on postoperative LLD. 34.5% of patients perceived LLD in the preoperative period, which decreased to 3.7% in the postoperative period. Perceived LLD did not correlate to radiographic LLD and functional outcome.

**Conclusions:**

There is no significant difference in radiographic LLD between unilateral and bilateral TKA. The functional outcome is adversely affected by radiographic LLD of ≥ 10 mm. Age, sex, BMI, preoperative LLD and difference in deformity angle do not affect the LLD. About one-third of patients perceive LLD in the preoperative period, which improves significantly after TKA.

**Levels of evidence:**

II.

## Introduction

Despite advancements in surgical techniques, implant design and better rehabilitation protocol, 15–20% of patients remain dissatisfied after total knee arthroplasty (TKA) [1, 2]. Although multiple factors are associated with poor functional outcome and dissatisfaction, limb length discrepancy (LLD) is a major concern that has been inadequately studied [3–5]. Unlike THA, the principle of TKA involves soft tissue balancing after appropriate bone resection to equalize the flexion-extension gap, and hence limb length alteration is unavoidable [5, 6]. Limb length discrepancy has been shown to increase the incidences of back pain, radiculopathy, gait disorders and general dissatisfaction [7–16]. The patient may perceive gait alteration after unilateral TKA as an unacceptable outcome. It has been proven that mechanical load and isometric torque are greater on the longer limb, which negatively impacts the nearby joints. Consequently, there may be aggravations of low back pain, hip pain, and compensatory pelvis and spinal curvature changes [7–16]. Such pathologies usually occur in LLD of 20 to 50 mm. Although shoe lift and insert can adjust the discrepancy up to 6 mm, its use after TKA may not be acceptable by the patients [11].

Only a few studies in the literature evaluated LLD after TKA [5, 6, 10, 14, 15, 17–23]. While the western literature reported minimal LLD (< 5.5 mm) that has no clinical relevance [6, 18–21, 23], others have reported substantial limb length discrepancy (≥ 10 mm) following unilateral TKA [10, 14, 15, 17]. The LLD issue is more alarming in India and few other Asian countries because of the late presentation, advanced disease with bone defect, ligamentous laxity and proceeding for unilateral TKA despite severe bilateral disease. As the osteoarthritis (OA) knee is usually bilateral, the patients try to adapt to the nonoperative limb by keeping the operated knee in a flexion attitude [24]. Over the long run, the operated knee may develop a flexion contracture, thus compromising the function. Vaidya *et al*. reported that the functional outcome was significantly affected after unilateral TKA in a bilateral OA knee because of LLD [15]. Similarly, Kim *et al*. reported that minimal postoperative LLD should be intended to improve the functional outcomes of primary TKA [4].

The preoperative LLD and perceived LLD in TKA have recently gained more importance [18–20]. However, many studies have not evaluated these aspects. Studies reported that, among patients, preoperative LLD incidence is higher than that of postoperative LLD, and most of the preoperative LLD settles after TKA [19]. The perceived LLD has shown no correlation to radiographic LLD. Again, the variables affecting radiographic LLD were not shown to influence perceived LLD [19]. With limited available literature, it is difficult to comment on the impact of radiographic and perceived LLD on functional outcome. Therefore, the present study was designed to assess the radiographic and perceived LLD between the legs in both preoperative and postoperative periods in unilateral and bilateral TKA. The impact of LLD on the functional outcome was evaluated, and factors affecting LLD were assessed. The secondary objectives were to look for the magnitude of limb length alteration in the ipsilateral limb following TKA and its correlation to the type and severity of the deformity.

## Materials and methods

### Study design and patient recruitment

In this prospective observational study, all primary OA knee patients (Kellegren Lawrence grade 3 or 4) who received unilateral or bilateral TKA in our institute between January 2019 and March 2020 were included for evaluation of limb length discrepancy. The decision to undergo a unilateral or bilateral procedure in these bilaterally-affected patients was based on their severity of symptoms and demand. Patients who were severely symptomatic on both sides, whose daily activities were affected and who had daily need of analgesics and severe night pain were considered for bilateral TKA. Excluded were the patients with flexion contracture of > 15º, previous surgery on the ipsilateral limb, hip pathology, severe ankle or foot deformity, severe extraarticular deformity requiring osteotomy, and inability to stand or walk. The patients were recruited after obtaining Institutional Ethical Committee approval (IEC/AIIMS BBSR/PG Thesis/2018–19/43). A total of 104 patients were included, of which 23 were lost to follow-up because of the COVID pandemic. The remaining 81 patients (involving 112 knees) who completed at least six months of the follow-up were finally assessed. There were 31 bilateral TKA and 50 unilateral TKA. Twenty-three bilateral TKAs were performed in one operative setting whereas the remaining 8 bilateral TKAs were performed in two settings in one hospitalization with a gap of seven days.

### Patient evaluation

The demographic profile of the patients, *i*.*e*., age, sex, height, weight and BMI, were entered into a pre-designed proforma. The clinical examination of the knee was performed, emphasizing the details of the pain, range of motion and the degree of varus/valgus deformity. The perceived LLD was evaluated by asking the patients if they felt a difference in the length of their legs. If the answer was "yes", it was considered that the patient could perceive the LLD. Preoperative radiographs of both knees (anteroposterior, lateral and skyline view) were evaluated for OA severity using Kellegren Lawrence (KL) grading. The standing lower limb scannography was done to measure the length of lower limbs, deformity and Hip-Knee-Ankle (HKA) angle in the preoperative period.

### Surgical procedure

The TKA was performed by a single surgeon (SKT) under spinal anesthesia after applying a tourniquet via a standard medial parapatellar approach. The principle of measured resection technique was adopted to achieve a neutral mechanical axis and appropriate ligament balancing. The posterior-stabilized cemented knee prosthesis was implanted uniformly under a bloodless condition, and the lumen of the femur was plugged with autologous bone. The bone resection was kept to minimal with the thinnest possible polyethylene insert. All patients were mobilized the next day of surgery, and a uniform physiotherapy protocol was adopted.

### Postoperative evaluation

**Fig. 1:**
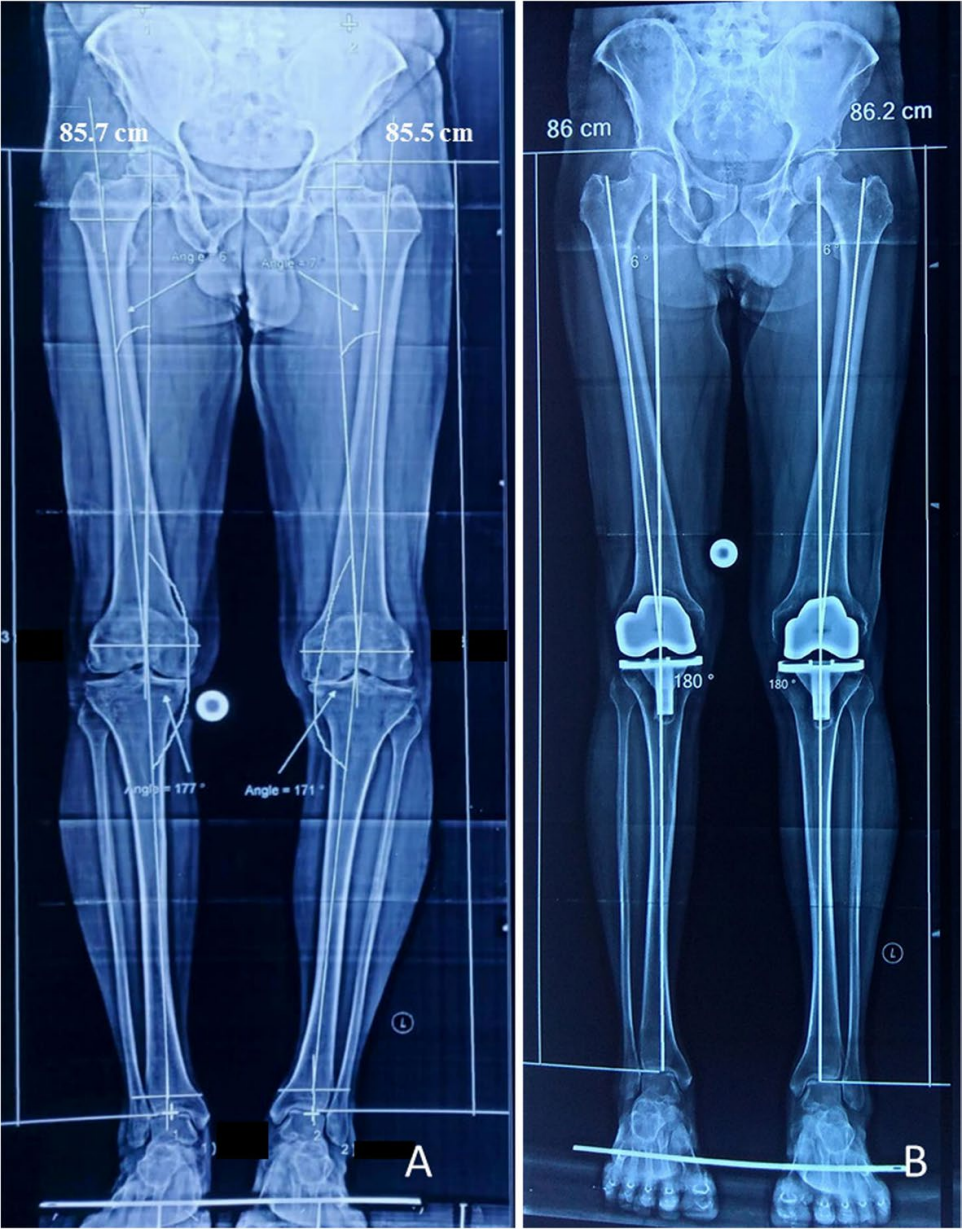
Preoperative (**A**) and postoperative (**B**) scannograms showing limb length variations in a 65-year-old male patient with bilateral OA knee and varus deformity; (**A**) Preoperative varus right, left: 3º, 7º, limb length right, left: 85.7 cm, 85.5 cm. (**B**) Postoperative scannogram shows 0º HKA angle on both sides, limb length right, left: 86 cm, 86.2 cm

The postoperative lower limb scannography was performed six weeks after the surgery for radiographic evaluation of leg length and angles. The HKA angle was measured using two intersecting lines on the film as described by Cooke *et al*. [25]. The mechanical axis of the femur was obtained by drawing a line intersecting the centre of the femoral head and the intercondylar notch of the distal femur. The tibial mechanical axis was obtained by drawing a line from the centre of the tibial plateau to the centre of the tibial plafond. The HKA was defined as the angle between these two axes. The radiographic limb length was measured using automated software as described by Lang *et al*. [21]. It is the distance between the top of the femoral head and the base of the tibial plafond in a full-limb radiograph (Fig. 1). The patients' functional outcome (Western Ontario and McMaster Universities Arthritis Index-WOMAC, minimum 0 and maximum 96 points) and perceived limb length discrepancy were evaluated at the end of six months [26].

The alteration of ipsilateral limb length and LLD between the legs were evaluated after assigning the patients into different groups (preoperative/postoperative, unilateral/bilateral, varus/valgus). First, the preoperative and postoperative limb lengths were determined and compared. The average LLD was compared between unilateral and bilateral TKA groups. The patients were divided based on preoperative limb alignment (valgus/varus); the average limb length differences in each group were determined and compared. The LLD in the varus deformity group was compared on the basis of the severity of the deformity, with ≥ 10º *vs.* < 10º. The variables affecting radiographic and perceived LLD were evaluated. Finally, the impacts of radiographic and perceived LLD on the functional outcome were evaluated.

### Statistical analysis

The statistical analysis was performed by using R-software. Descriptive statistics were used to describe the mean and standard deviation of the parameters. The mean differences in variables with continuous outcomes were analyzed using the independent *t* test. The preoperative and postoperative parameters were compared using paired *t* test. The Chi-square test and Fisher's exact test were used for categorical variables. The variables affecting LLD were analyzed with multivariate linear regression analysis. The statistical level of significance was set at *P* < 0.05. The sample size was estimated during the protocol submission to the ethics committee. For a single group test with SD of 1.1 (as obtained from the study of Tipton *et al*.), alpha error of 5% and power of 80%, the sample size was found to be 104 TKAs.

## Results

### Demographic details

The mean age of the patients was 60.33 ± 7.75 years (range, 46 to 75 years). There were 59 (72.8%) females and 22 (28.2%) males. The average height, weight and BMI of the patients were 156.47 cm ± 10.39 cm, 65.27 kg ± 12.64 kg and 26.62 ± 4.4 respectively. There were 107 varus knees and five valgus knees.

### Radiographic LLD

**Fig. 2:**
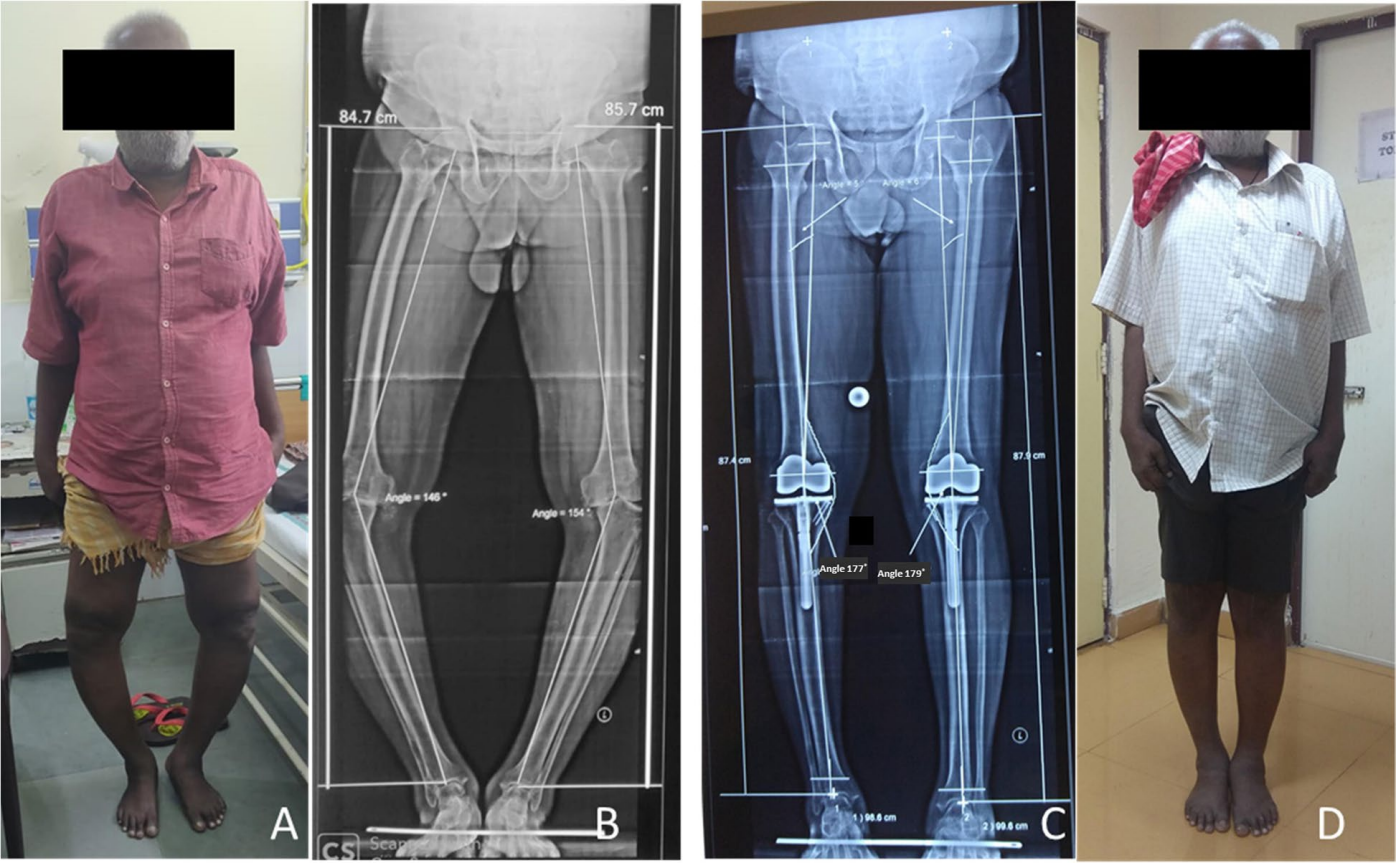
**A**, **B**, 60-year male with severe bilateral osteoarthritis knee and varus deformity (Right varus 34° and Left varus 26º, limb length right 84.7 cm, left 85.7 cm); **C**, **D**, Six weeks following bilateral TKA, the deformity in Rt knee decreased to 3° varus and left knee decreased to 1° varus (limb length Rt 87.4 cm, Lt 87.9 cm). The limb was lengthened by 2.7 cm and 2.2 cm on the right side and left side, respectively

**Fig. 3:**
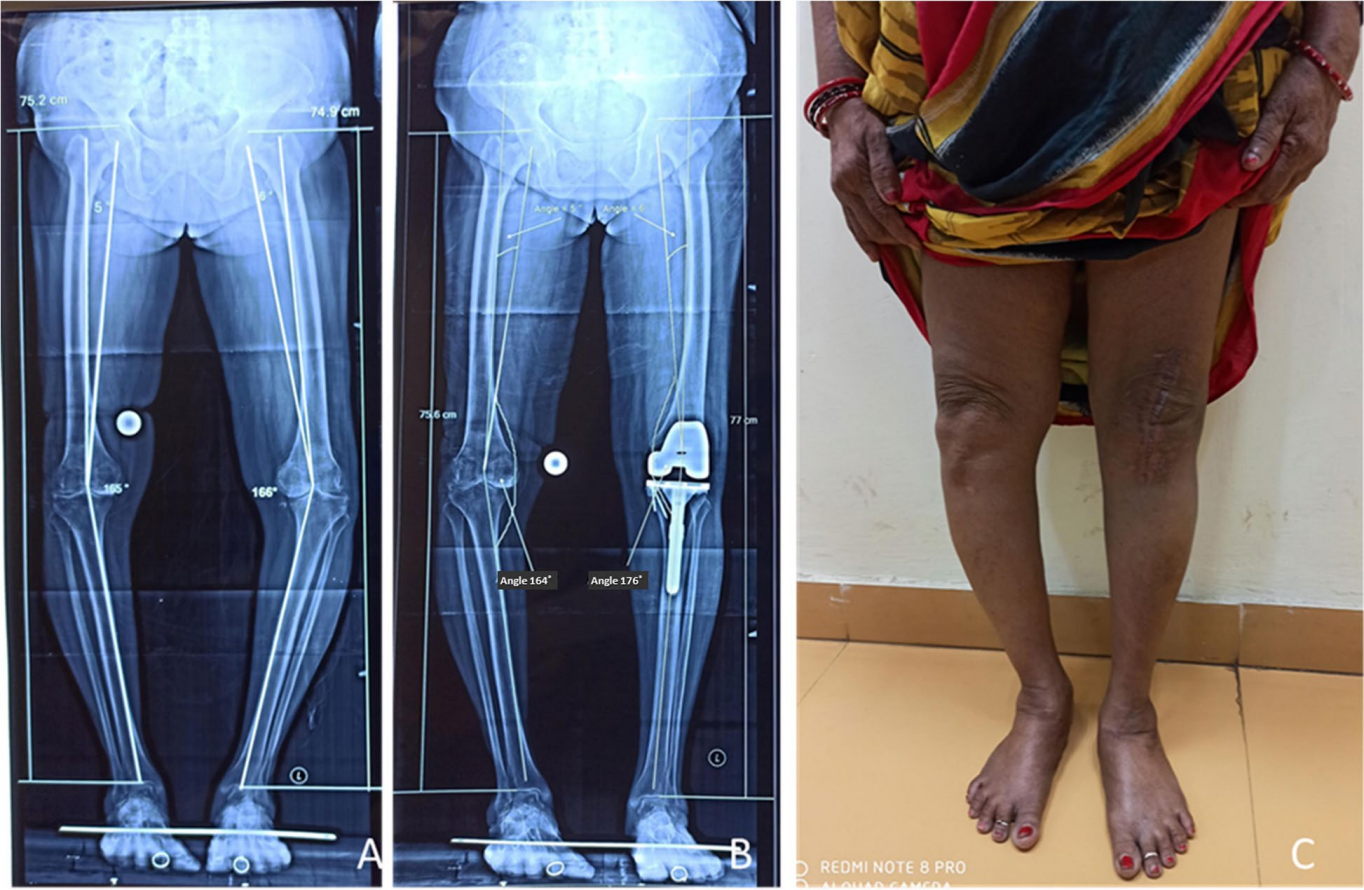
65-year female with bilateral varus deformity, right 15º varus and left 14° varus; Preoperative limb length, right 75.2 cm, left 74.9 cm. Postoperative limb alignment following left TKA, right 16º varus, left 4º; Postoperative limb length right 75.6 cms, left 77 cm. The clinical photograph shows a nearly straight leg on the left side, whereas the right limb still looks varus

Preoperative LLD in the unilateral and bilateral TKA groups was 0.75 cm ± 0.60 cm and 0.58 cm ± 0.52 cm respectively (*P* = 0.197, Table 1, Fig. 2 and 3). Similarly, postoperative LLD in the unilateral group was 0.76 cm ± 0.85 cm (Fig. 3) and in the bilateral group was 0.59 cm ± 0.92 cm (*P* = 0.402, Fig. 3). The postoperative LLD was < 10 mm in 65 patients (80.2%) and ≥ 10 mm in 16 (19.7%) patients.

**Table 1:** Ipsilateral limb length change and LLD between the legs in the study patients based on the severity of deformity and laterality of the procedure

Parameters	Limb length change (cm)	LLD (cm)	*P*-value
Varus	0.93 ± 0.91		< 0.001
Varus < 10	0.68 ± 0.08		< 0.001
Varus > 10	1.07 ± 1.17		< 0.001
Valgus	1.56 ± 0.89		< 0.017
Unilateral		0.76 ± 0.85	0.402
Bilateral		0.59 ± 0.92

A significant improvement in functional score was observed in all patients after TKA. The mean preoperative WOMAC score was 69.62 ± 11.15, and it was 13.40 ± 5.41 after six months (*P* < 0.001). When WOMAC score was correlated against LLD, there was a significant negative correlation between functional outcome and LLD of ≥ 10 mm **(**correlation coefficient = 0.54, *P* < 0.001), but no significant correlation was observed between functional outcome and LLD of < 10 mm (correlation coefficient = 0.083, *P* < 0.510). Linear regression analysis was performed to evaluate the effect of variables on postoperative LLD. There was no significant effect of age, sex, height, weight, BMI, preoperative LLD and preoperative difference in deformity between the limbs on postoperative LLD (Table 2). All patients in this study had a severe KL grade (≥ 3), so the effect of severity of OA could not be correlated to LLD.

**Table 2:** Regression analysis of LLD with variables like age, sex, height, weight, BMI, preoperative LLD and difference in preoperative deformity between the legs

Variables	LLD		
	Estimate	Standard error	*P*-value
Age	–0.002	0.014	0.837
Sex	0.41	0.32	0.203
Height	0.004	0.80	0.957
Weight	–0.20	0.097	0.836
BMI	0.043	0.232	0.851
Preop LLD	0.108	0.212	0.610
Difference in preoperative deformity	–0.0199	0.021	0.354

The residual standard error is 0.902, R-squared:–0.052, the *P*-value of 0.878

### Perceived LLD

34.5% (*n* = 28) of the patients could perceive LLD in the preoperative period. However, only 3.7% (*n* = 3, with 2 receiving unilateral TKA, and 1 receiving bilateral TKA) of the patients perceived the LLD in the postoperative period. There was no correlation between perceived LLD and radiographic LLD in the preoperative (correlation coefficient = 0.17) and postoperative periods (correlation coefficient = 0.11). The patients who perceived LLD in the preoperative period had a mean WOMAC score of 60 compared to 66 in patients who did not perceive it (unpaired *t* test, *P* = 0.3). Similarly, patients who perceived LLD in the postoperative period had a mean WOMAC score of 14. The postoperative WOMAC score in patients who did not perceive LLD was 12.8. This difference was not significant (unpaired *t* test, *P* = 0.41). There was no correlation between perceived LLD and WOMAC score (correlation coefficient = 0.09).

### Ipsilateral limb length alteration

The average limb length alteration was 0.92 cm ± 0.98 cm and it was statistically significant (*P* < 0.001, paired *t* test). The limb got lengthened in 91.1% (*n* = 102) of the patients after TKA (mean lengthening of 0.67 cm). However, there was no limb shortening in any patient, and 8.9% (*n* = 10) of the patients experienced no change in limb length.

The preoperative varus angle of 13.18 ± 6.93 degrees was corrected to 2.38 ± 2.36 degrees after TKA (*P* < 0.001). The overall limb lengthening in the varus knee was 0.93 cm ± 0.91 cm (*P* < 0.001). Among patients with < 10 degrees of varus (*n* = 40), the limb length alteration was 0.68 cm ± 0.08 cm (*P* < 0.001, Fig. 1). Similarly, the change in limb length was significant in patients with varus deformity of ≥ 10 degrees (*n* = 67, 1.07 cm ± 1.17 cm, *P* < 0.001) (Table 1, Fig. 2). The limb length alteration in patients with varus deformity of ≥ 10 degrees was significantly greater than in patients with < 10 degrees (*P* = 0.02).

In the valgus knee (*n* = 5), the mean preoperative valgus of 16.38 ± 7.76 degrees was corrected to 4.00 ± 2.13 degrees (*P* < 0.001). The mean limb length alteration was 1.56 cm ± 0.89 cm, (*P* = 0.017) (Table 1). There was a significantly increased limb lengthening following TKA in valgus knee compared to varus knee (mean difference = 0.605 cm, *P* = 0.003).

## Discussion

This study revealed that LLD of ≥ 10 mm following TKA significantly affected the functional outcome. Age, sex, height, weight, BMI, preoperative LLD and difference in deformity between the limbs did not affect the LLD. The limb usually got lengthened after TKA compared to its preoperative state. However, the extent of limb lengthening was significantly greater in the valgus knee compared to varus.

The incidence of limb lengthening after TKA in the literature varies between 44% and 83.3% [5]. The studies by Lang *et* *al*. and Tipton *et al*. reported an average limb lengthening of 6.3 mm and 4.38 mm, respectively [21, 23]. However, about 91% of patients reported a limb lengthening in our study, and the average limb lengthening was also higher (0.92 cm ± 0.98 cm). The reason for a higher incidence and extent of limb lengthening in this study was the inclusion of patients with advanced disease (KL grade ≥ 3) and more severe deformity. The mean preoperative varus angle in this study was 13.18 degrees, and the amount of angular correction after TKA was 11 degrees. However, there was only a 6.5-degree and 4.48-degree change in alignment in the series of Lang *et al*. and Tipton *et al*. [21, 23]. It is apparent that the limb length change in the operated limb depends on the severity of preoperative deformity and the extent of correction [10]. Khalifa *et al*. predicted a limb length change of 4 mm for each 10-degree correction in HKA angle [10]. Accordingly, the more limb length change is expected in the developing and underdeveloped countries where the patients often visit at an advanced stage with extremes of deformity [14].

The reason for more limb length alteration after valgus knee TKA is still not apparent, but consistently, all studies reported a higher limb lengthening in the valgus-deformed knee compared to varus deformity [5, 21, 23]. Probably, the inflammatory nature of the disease in the valgus knee allows for more stretching of the ligaments during surgery. A pooled analysis of two studies in the previously published meta-analysis reported a limb lengthening of 7.06 mm in the valgus knee compared to 4.42 in the varus knee (*P* = 0.03) [5].

Primary osteoarthritis of the knee often occurs bilaterally. Accordingly, the functional outcome is affected by the LLD between legs and not merely by the ipsilateral limb length alteration. Sabir *et al*. warned that unilateral TKA in bilaterally-affected OA knee might end up with significant LLD causing patient dissatisfaction and poor functional outcome [14]. In agreement with previous literature, this study also found that the functional outcome was adversely affected when LLD exceeded 10 mm. Kim *et al*. reported a significantly worse functional outcome in terms of stair climbing and Knee Society Score in patients with a postoperative LLD of ≥ 15 mm [4]. However, Chinnappa *et al*. found no significant correlation between radiographic LLD of ≥ 10 mm and functional outcome [18]. Only 11% of patients in their series had radiographic LLD of ≥ 10 mm. They reported perceived LLD as the major determinant of outcome.

A few studies have reported that radiographic LLD and perceived LLD existed in the preoperative period in unilateral OA knee [18–20]. These two parameters improved after TKA. The authors of these studies suggested that the patients should be informed of the existence of such LLD in the preoperative period [18–20]. We tried to look for the existence of similar preoperative LLD in our patients who had bilateral disease. We found that the preoperative LLD could be found in bilateral OA knee because the severity of deformities was different in both knees. Recently, the perceived postoperative LLD or the awareness of limb length discrepancy among patients is increasingly accepted by the researchers [5]. No association was found between radiographic LLD and mechanical HKA angles. Goldstein *et al*. observed 25% preoperatively perceived LLD and only 10% was in the postoperative period. Of 18 patients (25%) with preoperative LLD in their series, only one had persistently perceived LLD in the postoperative period. They concluded that most of the preoperative LLD gets settled with surgery [19]. In patients with persistent or newly developed postoperative LLD, complete resolution was noted within three months. Our findings were similar to that of Goldstein *et al.* [19]. There were 34% perceived LLD in the preoperative period and only 3.7% LLD in the postoperative period. Only two patients in the unilateral group (*n* = 50) and one in the bilateral group (*n* = 31) perceived LLD in the postoperative period. There was no correlation between radiographic and perceived LLD in this study and this result was contradictory to the finding of Vaidya *et al*. [15] and Sabir *et al*. [14], who reported perceived LLD in patients with a radiographic LLD of more than 2 cm. However, Goldstein *et al*. reported no effects of variables like age, sex, BMI and mechanical alignment on perceived LLD [19]. Likewise, this study failed to show that functional outcome was affected by perceived LLD.

There are several factors that affect LLD after TKA [5, 15, 17–21, 23]. Age and gender were not found to affect the LLD in multiple previous studies [17, 19–21]. One study reported a significant association between perceived LLD and the female gender [18]. We did not observe any impact of age and sex on LLD or limb length change. The effect of BMI on LLD is difficult to comment, as a few studies omitted morbid obese patients. Chang *et al*. reported the association between greater LLD and smaller BMI in their series of 466 patients at one-year follow-up [17]. However, there was a small percentage of patients (12.3%, *n* = 10) in our study who were obese, and we could not find any effect of BMI on LLD. The meta-analysis on LLD in TKA did not find a significant difference in the preoperative and postoperative LLD (MD = –1.23, 95% CI: –3.72, 1.27, *P* = 0.34) [5]. Our study had similar observations. Linear regression analysis did not find any association of age, sex, height, weight, BMI, preoperative LLD and preoperative difference in deformity with postoperative LLD. The severity of knee osteoarthritis may also play a role [5]. However, Lange *et al*. found that even with Kellegren and Lawrence (KL) grade > 2 in the non-operated limb, there was no considerable difference in the postoperative leg length between the operated and non-operated limbs [21]. In agreement with the literature, despite having grade 3 or 4 OA on both knees of the patients in this study, we did not observe a significant difference in LLD between the operated and non-operated limbs in unilateral TKA patients in preoperative and postoperative periods. Similar findings were noted in bilateral TKA patients as well.

While four studies reported a smaller radiographic LLD in bilateral TKA than in unilateral TKA [14, 17, 21, 23], the difference was not significant. The assessment of pooled data did not show a statistically significant difference between unilateral and bilateral cases either [5]. A similar observation was noted in our study (mean difference = 0.16 ± 0.2, *P* = 0.402). However, Vaidya *et al*. and Sabir *et al*. had a unique finding [14, 15]. They reported higher incidence of perceived LLD among unilateral TKA patients who had radiographic LLD of > 2 cm. Such a substantial radiographic LLD and perceived LLD among the unilateral TKA patients in their series were seen in those unilateral TKA patients who were ideal candidates for bilateral TKA but refused to undergo bilateral procedure due to a financial or personal problem. However, the patients in our series underwent unilateral or bilateral TKA based on their demand. The unilateral TKA patients had contralateral OA knee with a radiographic grade of 3 or 4, but it was not symptomatic enough to demand a replacement surgery.

There were certain limitations in this study. The lateral images were not used in this study to assess changes in limb length, and only a standing AP scannogram was used to measure limb length and alignment. Thus, the deformity other than the coronal plane may have had a role and gone unrecognized. The inter-observer and intra-observer variability in measuring the radiographic LLD was not assessed. However, as we used digital-based measurement employing software to mark the points, the chances of error were minimal. The flexion contracture preoperatively may have affected limb length measurement. However, one study suggested that limb length measurement was not affected by flexion contracture if it was less than 15° [27]. Identification of the pertinent landmarks may occasionally be found to be difficult. The contrast of the film was manipulated digitally to compensate for over-penetration of the lower extremities for better identification of the landmarks. The strength of the study includes the inclusion of advanced OA knee, where the probability of LLD is higher after surgery. Again, the radiographic limb length was measured six weeks after TKA when the pain was minimal, and there was usually no residual flexion contracture [5].

## Conclusions

The functional outcome is adversely affected by radiographic LLD of ≥ 10 mm following TKA. Age, sex, BMI, weight, height, preoperative LLD and difference in deformity between the limbs do not affect the LLD. There is no difference in the preoperative and postoperative limb length discrepancy in the unilateral and bilateral TKA. About one-third of patients perceive LLD in the preoperative period, which improves significantly in the postoperative period. The limbs usually get lengthened following TKA compared to their preoperative state. The extent of lengthening in the valgus knee is greater compared to varus.

## Data Availability

The data of the study are available for sharing.
